# Deep winter convection and phytoplankton dynamics in the NW Mediterranean Sea under present climate and future (horizon 2030) scenarios

**DOI:** 10.1038/s41598-018-24965-0

**Published:** 2018-04-26

**Authors:** Diego Macias, Elisa Garcia-Gorriz, Adolf Stips

**Affiliations:** European Commission, Joint Research Centre, Directorate D- Sustainable Resources, Via E. Fermi, 21027 Ispra, VA Italy

## Abstract

Deep water convection (DC) in winter is one of the major processes driving open-ocean primary productivity in the Northwestern Mediterranean Sea. DC is highly variable in time, depending on the specific conditions (stratification, circulation and ocean-atmosphere interactions) of each specific winter. This variability also drives the interannual oscillations of open-ocean primary productivity in this important region for many commercially-important fish species. We use a coupled model system to 1) understand to what extent DC impacts phytoplankton seasonality in the present-day and 2) to explore potential changes in future scenarios (~2030). Our model represents quite accurately the present-day characteristics of DC and its importance for open-ocean phytoplankton blooms. However, for the future scenarios the importance of deep nutrients in fertilizing the euphotic layer of the NW Mediterranean decreases. The model simulates changes in surface density and on the levels of kinetic energy that make mesoscale activity associated with horizontal currents to become a more important fertilization mechanism, inducing subsequently phenological changes in seasonal plankton cycles. Because of our focus on the open-sea, an exact quantification of the impact of those changes on the overall biological production of the NW Mediterranean cannot be made at the moment.

## Introduction

Among the general oligotrophy of the Mediterranean Sea^1,2^ its north-western (NW) region presents mesotrophic conditions^3^ linked with several sources of nutrients to the surface layer such as the Atlantic influx, riverine discharge, atmospheric deposition and deep ocean convection^4^.

Deep convection (DC) is a winter phenomenon that transforms surface waters into intermediate and deep waters in three main regions within the Mediterranean Sea: the NW Mediterranean^5^, the southern Adriatic Sea^6^ and the Crete region^7^. The transformation of surface into deeper water masses partially drives the overall circulation of the Mediterranean basin^8^ and controls the main interchange with the open ocean through the Strait of Gibraltar^9^.

In the NW Mediterranean, the prevailing cyclonic circulation at the Gulf of Lion region^10,11^ provokes a doming of the isopycnals that weakens the vertical stratification^12^ and isolates the water masses within. DC is then triggered when cold and dry local winds blow from the north or from the north-west (*Mistral*, *Tramontana*)^13^, inducing an increase of the surface layer density by provoking strong evaporation and cooling. Henceforth, DC in the NW Mediterranean is controlled by a combination of the oceanographic patterns in the area and the strong local winds^14^.

**Figure 1:**
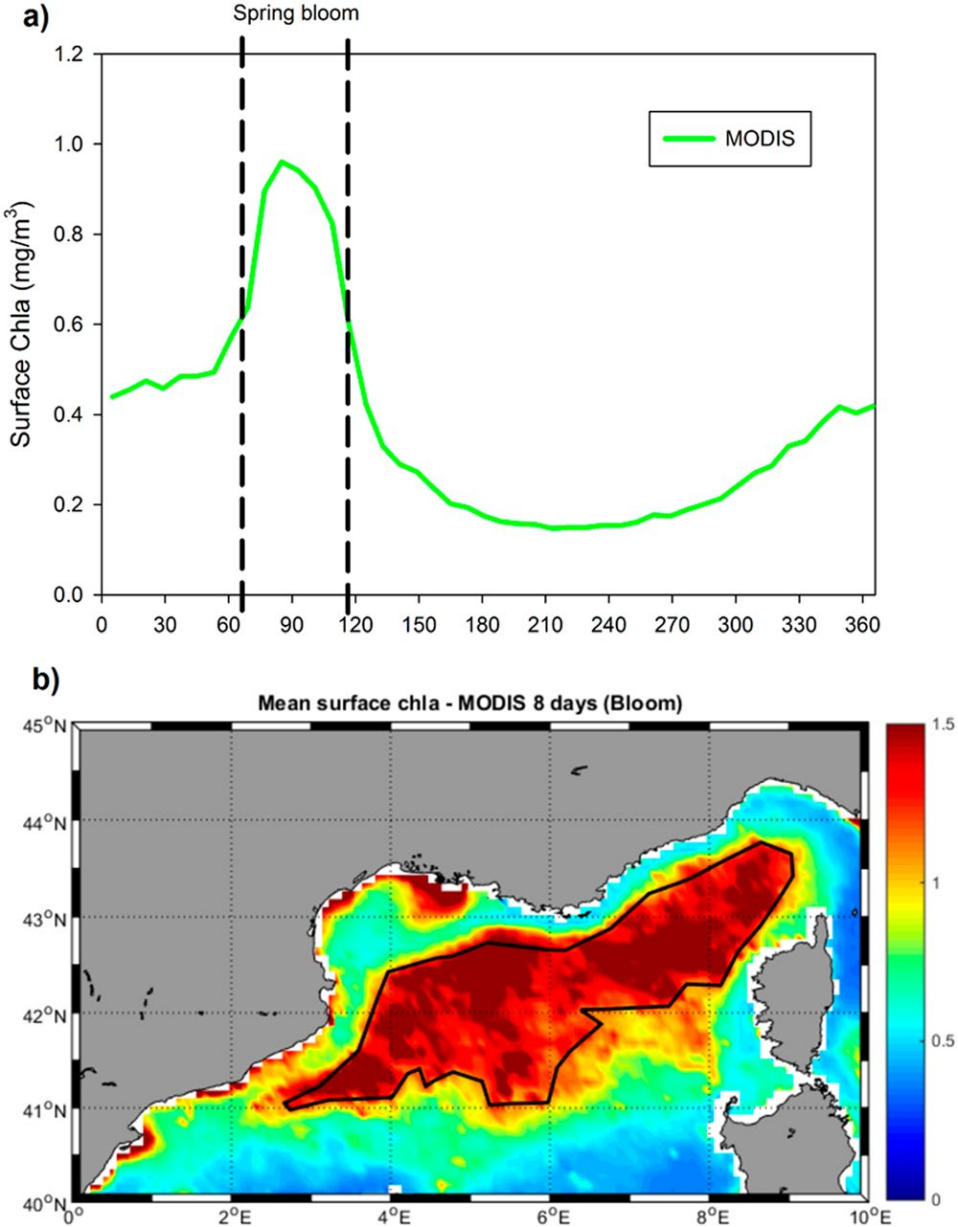
(**a**) Mean climatological surface chlorophyll-*a* (Chla) concentration (mg m^−3^) in the NW Mediterranean (see text for definition) from MODIS data (2003–2015). (**b**) Mean surface Chla (mg m^−3^) spatial distribution for the blooming period (vertical bars in panel a) from MODIS data (2003–2015). Maps were created by the authors using MATLAB software vR2014b (https://it.mathworks.com/products/matlab/matlab-graphics.html).

DC events can be an important source of nutrients for the surface layer of the NW Mediterranean^15^ and the nitrate and phosphate levels observed at surface during convective episodes can be very close to deep concentrations^16,17^. These nutrients inputs are larger than terrestrial^18^ and atmospheric^19^ inputs and could drive the strong seasonal cycle of phytoplankton observed in this region. Here, a typical seasonal cycle of phytoplankton biomass comprises a strong winter/spring bloom followed by summer oligotrophy and a less intense secondary bloom in autumn^20–22^ (Fig. 1a).

The large nutrients input to the euphotic layer during winter DC has been described to be a major trigger of the spring, open-ocean phytoplankton bloom typical of this area^23–28^. Also the plankton community structure^29^ and the phytoplankton surface abundance and horizontal distribution in spring^30^ seem to be controlled by the duration, intensity and extension of the winter DC in the NW Mediterranean region^31^.

Although DC is an annual event in the NW Mediterranean, it presents a high degree of interannual variability concerning its duration and spatial extension^32–34^ as it depends on each year’s particular atmosphere-ocean interactions (cooling against stratification)^4,35,36^. It has been proposed that this high variability of DC could lead to interannual variability of the intensity and spatial extension of the associated phytoplankton spring blooms^37–41^.

However, it is quite challenging to study *in situ* DC events and their associated biogeochemical effects due to their occurrence during winter months, its spatial extension and its relatively short duration^4,42^. Some exceptional recent efforts have applied a multidisciplinary approach to study DC in the NW Mediterranean as, for example, the MERMEX (Marine Ecosystems Response in the Mediterranean Experiment) project^43^. In this investigation, field-based measures^30^, remote sensing and autonomous observation platforms^44^ were combined with numerical models^43,45^ to extensively characterize the 2012–2013 DC event.

Nevertheless, given the difficulties to observe directly this process (either through field-based measures or using remote sensing), numerical models are one of the most common approaches to study DC in the NW Mediterranean. Modelling efforts started in the 80 s by creating 1D representations of the vertical dynamics of the water column^32^ and continued in the following decade with the first 3D models^46–48^. More recently, improved models in terms of spatial and temporal resolution, atmosphere-ocean interactions and water masses initialization have allowed to more accurately represent specific DC events^43,49–52^ and, finally, to reproduce multi-annual variability in DC^34,36,53,54^.

The majority of previous modelling efforts have been centred on the physical description of the DC with only a handful of works considering the potential effects on the biogeochemistry of the region^29,55–57^. Here we use a coupled, regional earth system model (RESM) that includes the atmosphere, the rivers and the ocean (hydrodynamics + biogeochemistry) developed at the Joint Research Centre of the European Commission^57^. This modelling framework (MF) has been used to represent present^58,59^ and past^60^ hydrodynamic and biogeochemical conditions of the Mediterranean basin as well as to explore future conditions of its marine ecosystems^61^. This MF has been designed to analyze scenarios for the environmental status of Mediterranean ecosystems^62^ under different climatic conditions and, specially, considering alternative management (policy) options^63^. In previous applications^9^ the MF has been shown to be able to appropriately represent the position, seasonal cycle and intensity of main winter DC events in the Mediterranean Sea, although the relatively low spatial resolution of the ocean model (~9 × 9 km, see details in methods) prevents the correct representation of some DC events happening very close to the coast (such as in the northern Adriatic Sea).

In the present contribution we first evaluate the capability of the MF to represent current conditions of DC and associated chlorophyll-*a* (Chla) blooms by performing a hindcast run covering the period 2000–2015 and comparing model results with available remote sensing information. Afterwards we analyze a set of future scenarios for the year 2030 under different emission scenarios simulated by two different global circulation models (GCMs) included in the latest report of the Intergovernmental Panel for Climate Change (IPCC). In these scenarios the strength of DC and its link with the marine productivity are evaluated against the results obtained from the hindcast simulations.

## Results

We have divided the presentation of the results in two main subsections. In the first one model performance in terms of present day conditions in the NW Mediterranean Sea (defined as the region between 40°N–45°N and 0°E–10°E) is evaluated against remote sensing information. In the second section, the changes in hydrodynamic and biogeochemical conditions of the region for the four different future scenarios are analyzed.

### Present day model evaluation

Using the 13 years of MODIS (Moderate Resolution Imaging Spectroradiometer) 8 days data (2003–2015) the mean climatological surface Chla cycle of the NW Mediterranean (as defined above) was computed in Fig. 1a. The typical cycle for this region with a main bloom in spring and a secondary bloom in autumn^3^ could be observed. We use this cycle to define a ‘spring bloom period’ from Julian day 63 to Julian day 119 (vertical dashed lines in Fig. 1a) as the time when surface Chla concentration is more than twice its background value during the stratification period.

To understand where the Chla is found during this ‘*spring bloom’*, the mean map for that period during the 13 years is presented in Fig. 1b. It can be seen that there is an open-ocean bloom quite well separated from the coastal region and reaching values over 1 mg/m^3^. We use, precisely, this Chla value (which represents percentile 93 of all data) as threshold to define our ‘*blooming area’* (black contour in Fig. 1b). The comparisons between satellite-derived Chla and model-simulated Chla presented below are made using the data coming from this particular area of the NW Mediterranean.

A criterion for defining the mixed layer depth (MLD) was stablished on the basis of potential density differences. In our case we choose the threshold of 0.1 kg/m^3^ difference with respect to the surface value to define the base of the ML. Different values of potential density differences have been used in the literature to define the MLD^36,45^ or even different variables, as turbulent kinetic energy^53^. This diversity could lead to inconsistencies^35^ and make results from different works hard to compare^30^. However, this issue is of relatively lower importance for our analysis; we are more interested in making comparisons between different years/periods, so that should not be largely affected by the specific value for the threshold chosen.

**Figure 2:**
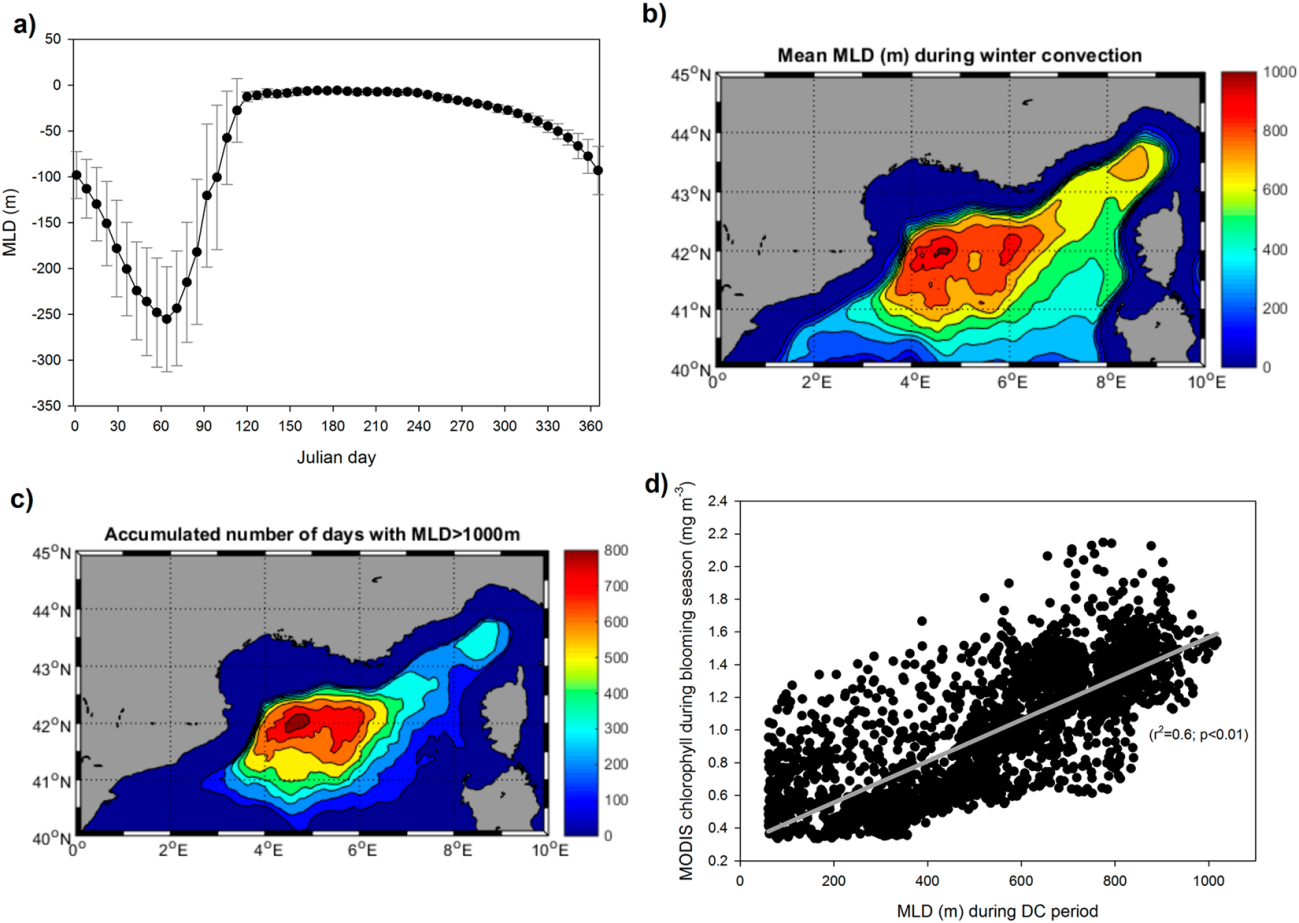
(**a**) Climatological seasonal mixed layer depth (m) on the NW Mediterranean (see text for definition) computed by the model for the 2000–2015 period. Black circles mean daily value, gray lines standard deviation range. (**b**) Mean mixed layer depth (m) during the convective period (from day 18 to day 110) during the simulation period (2000–2015). (**c**) Accumulated number of days during the simulation period (2000–2015) in which the mixed layer depth exceed the 1000 m threshold. (**d**) Scatter plot of the mean mixed layer depth distribution (panel b) versus the mean MODIS surface chlorophyll map during the blooming period (Fig. 1b). Maps were created by the authors using MATLAB software vR2014b (https://it.mathworks.com/products/matlab/matlab-graphics.html).

The performance of the model simulations in terms of depth of the ML, position of the deep convection zone and the frequency of apparition of MLD > 1000 m is evaluated in Fig. 2a–c. For the whole NW Mediterranean, the mean MLD shows a clear seasonal cycle (Fig. 2a) with maximum values (~250 m) towards the end of February (day 60) and a sharp shoaling afterwards reaching minimum depths of <5 m during the stratification period. A slight increase in MLD is observed during autumn (from day 270 onwards). The standard deviation of the mean MLD is larger during the autumn-winter month (gray lines in Fig. 2a) and much smaller during the stratification period.

If the mean MLD in the area is computed during the ‘convective’ period (defined as Julian day 18 to Julian day 110 according to Fig. 2a) a familiar distribution emerges (Fig. 2b) with larger MLD in the Gulf of Lion region (centered at ~42°N, 5°E)^64^ but also extending northwestward towards the Ligurian Sea^65^. A similar distribution could be seen, when the number of days with MLD larger than 1000 m during the 16 years of simulation are plotted (Fig. 2c). This figure indicates that DC is more frequent in the central region of the Gulf of Lion (average ~ 50 days/y) and less common in the Ligurian Sea (~20 days/y).

The spatial distribution of the mean MLD (Fig. 2b) resembles the mean surface Chla concentration during the spring bloom (Fig. 1b). Indeed, if the scatter of mean MLD during the convective period versus mean surface Chla during the blooming period is plotted (Fig. 2d), a significant direct correlation (r^2^ = 0.6) could be computed. This strong correlation seems to indicate that the DC events could be, indeed, responsible for the development of the spring bloom in the NW Mediterranean Sea.

**Figure 3:**
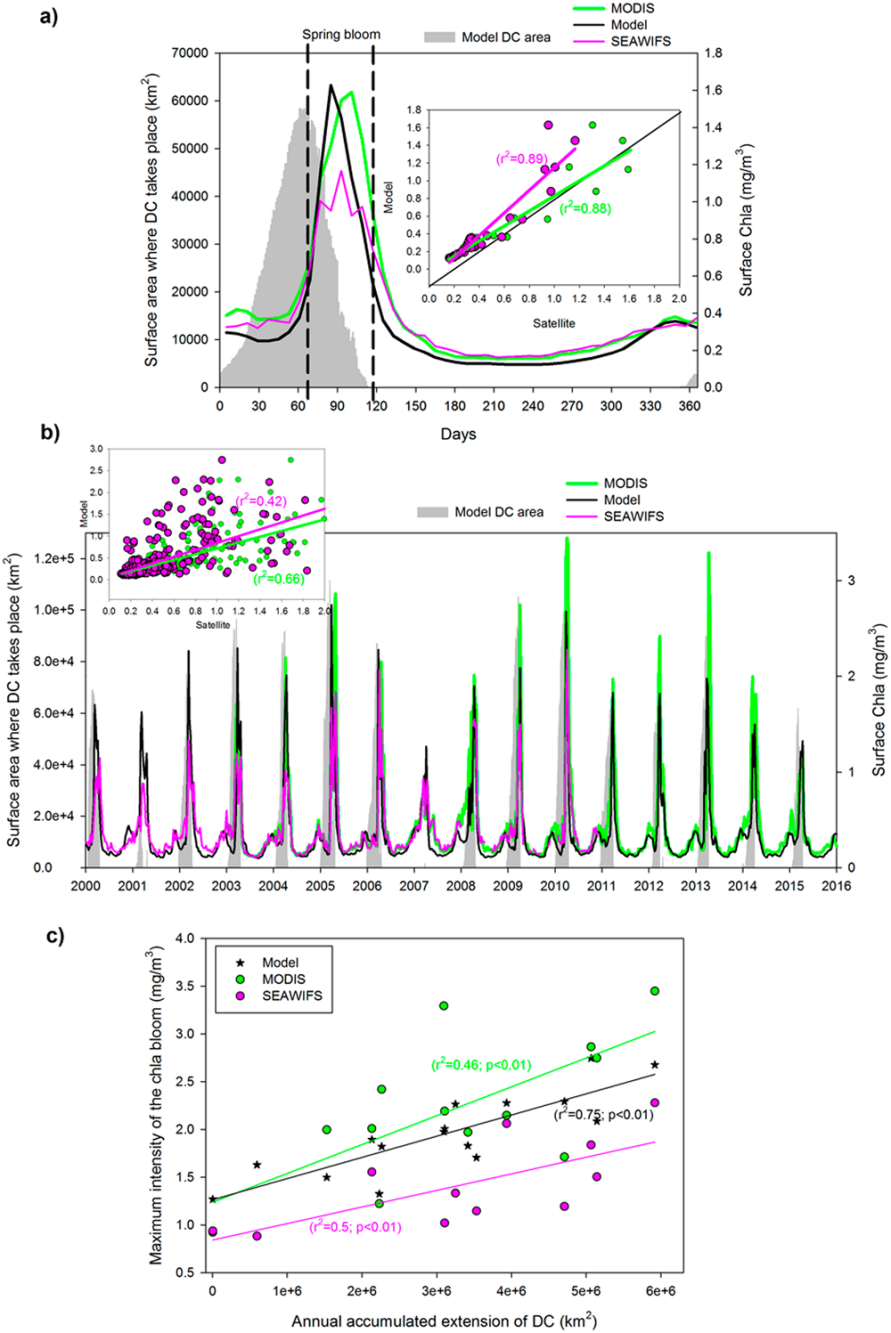
(**a**) Climatologic seasonal cycles of surface area where DC takes place (gray bars), model simulated surface chlorophyll-*a* (Chla) (black line), MODIS surface Chla (green line) and SeaWiFs Chla (magenta line). The inset show the scatter between the climatological values of model-computed Chla and the two different satellite-derived Chla products. (**b**) Time series of surface area where DC takes place (gray bars), model surface Chla (black line), MODIS surface Chla (green line) and SeaWiFs Chla (magenta line) for the period 2000–2015. The inset show the scatter between the model-computed Chla and the two different satellite-derived Chla products. (**c**) Scatter plot of the annual accumulated extension of the deep convection area (defined as the region where mixed layer depth > 1000 m) and the magnitude of the Chla bloom in the model (black symbols and line), MODIS (green symbols and line) and SeaWiFs (magenta symbols and line).

To further explore this possible link, Fig. 3a shows the climatological cycle of the area where DC takes place (gray bars) and the different climatological surface Chla cycles from satellite (both SeaWiFS and MODIS) and from the model (these Chla values are computed in the ‘*blooming area’* defined in Fig. 1b). The first thing we can notice from this Fig. 3a is that all Chla products are quite consistent, showing very similar timing and magnitude of the spring bloom and comparable values during the summer-autumn period. Indeed, the correlation between satellite-derived Chla (both SeaWiFS and MODIS) and the model-simulated Chla is quite good (r^2^ = 0.89 and r^2^ = 0.88 respectively) as shown in the inlet figure. The second pattern to be noticed is that the Chla bloom happens immediately after the maximum in DC (bear in mind that both phenomena take place in almost the same area as shown by Fig. 2d).

The interannual variability of both DC and spring bloom could be observed in the time-series of Fig. 3b. Again, satellite estimations and model computations of surface Chla show a reasonable correlation (r^2^ = 0.42 for SeaWiFS and r^2^ = 0.66 for MODIS) using the 8 days composite for the whole 16 years simulation period (inlet in Fig. 3b). Observing the time-series in this figure, certain coherence could be seen between the magnitude of the DC in a certain year and the maximum value of the bloom for that year. For example years 2001 and 2007 show almost no DC and very reduced spring bloom. On the contrary years 2005, 2010 and 2013 show much larger DC and unusually high spring blooms. Moreover, the different time-series show a long-term linear trend (Table 1). Two of the three Chla series (MODIS and model) show a decreasing trend in bloom intensity, which is matched by a decreasing trend on the extension of the DC area. SeaWiFS, on the contrary, shows a positive trend in the surface Chla concentration, though SeaWiFS has no data available from 2011 onwards in the NASA webpage.

**Table 1:** Linear trends computed from the time series shown in Fig. 3b.

Variable	Time period	Rate of change (p-value)
SeaWiFS Chla	(2000–2010)	+6.7e^−3^ mg/y (0.07)
MODIS Chla	(2003–2015)	−3.3e^−3^ mg/y (0.49)
Model Chla	(2000–2015)	−5.0e^−3^ mg/y (0.09)
Model DC area	(2000–2015)	−2.1e^+2^ km/y (2.59e^−04^)

To further confirm this correlation between DC and phytoplankton bloom at interannual scale, Fig. 3c shows the scatter of the accumulated extension of DC (i.e., the total area where in a given year MLD reached over 1000 m) and the maximum value of the surface Chla in the ‘*blooming area’* (as defined in Fig. 1b). For both satellite products and for the model simulations there is a significant relationship between the extension of the DC and the magnitude of the spring bloom (all correlation shown are significant to 99%).

### Future (2030) scenarios for the NW Mediterranean

Before evaluating the conditions of the NW Mediterranean in the future scenarios, we need to test if present-day conditions in the region are well simulated by our ocean model when forced with the different RCM-GCMs (Regional Climate Models – Global Circulation Models) combinations for the ‘evaluation period’ (*i*.*e*., 1998–2005). A previous work^66^ demonstrated that, after bias correcting the CCLM (Cosmo-Climate Limited Area Modeling) RCM forcing for the evaluation period, surface conditions were reasonably reproduced for the entire Mediterranean by the MF. However, the focus of that previous work was not specifically the NW region or the deep water formation process. In order to make sure that the RCM-GCMs forced runs provide similar conditions in the area of interest during the present climate, Fig. S1a shows the climatological SST (sea surface temperature) cycle in the deep convection zone (DCZ) (defined as the region with mean MLD of 650 m in Fig. 2b) for the runs using ERAin (the European Centre for Medium-Range Weather Forecasts database) forcing and CCLM-EcEarth and CCLM-MPI forcings (EcEarth and MPI being two of the GCMs included in the latest IPCC reports, see details in methods). In all cases SST anomalies are very small (average difference for the entire year ~0.27 °C for MPI and ~−0.19 °C for EcEarth) and, especially for the winter months (0.05 °C and 0.014 °C respectively) when DC is taking place. Moreover if the isotherm 13.7 °C (the average winter temperature in the DCZ for the three model runs) is plotted (color lines in Fig. S1b), a very good agreement with the location of deep mixing from Fig. 2b is found (black line in Fig. S1b). The comparisons shown in Fig. S1 indicate, henceforth, that the ocean simulations forced by the atmospheric conditions derived from CCLM-GCMs allow a reasonable representation of the NW Mediterranean conditions for the present-day climate.

**Figure 4:**
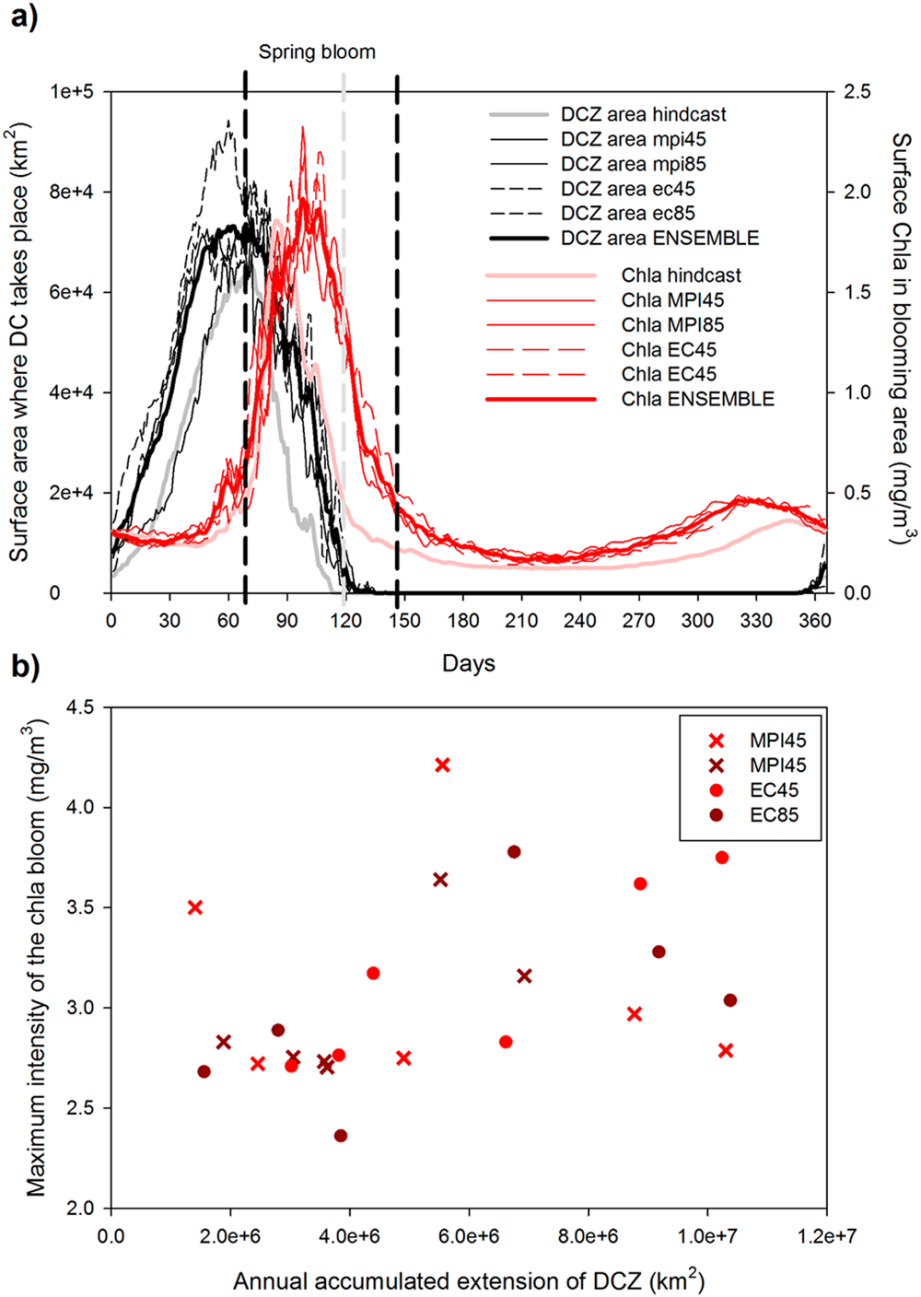
(**a**) Climatologic seasonal cycles of mixed layer depth and model simulated surface chlorophyll-*a* (Chla) for the hindcast period (gray and light red lines) and for the ENSEMBLE mean in the forecasts (black and red lines). The individual members’ simulations for the forecast are included as thin discontinuous lines. (**b**) Scatter plot of the annual accumulated extension of the deep convection area (defined as the region where mixed layer depth > 1000 m) and the magnitude of the Chla bloom in the different scenarios’ simulations.

Regarding the scenario simulations, Fig. 4a shows the comparison between actual and future climatogical cycles of DC area and surface Chla bloom. Regarding the DC, in the future (black lines, Fig. 4a) DC starts earlier and lasts longer than in the present (gray line, Fig. 4a). Also the maximum extension where DC takes place is larger in the future. The different members of the ENSEMBLE (thin black lines) present a fairly small variance around the mean value (thick black line).

Future surface Chla blooms (dark red lines, Fig. 4a) are larger and happen later in the year than the present day one (light red line, Fig. 4a). Also a small inter-ENSEMBLE variability is found (thin dark red lines) as happened with the DC area. During the stratified period, all scenarios show larger Chla level with respect to the actual values with the autumn bloom happening earlier (~30 days before).

**Figure 5:**
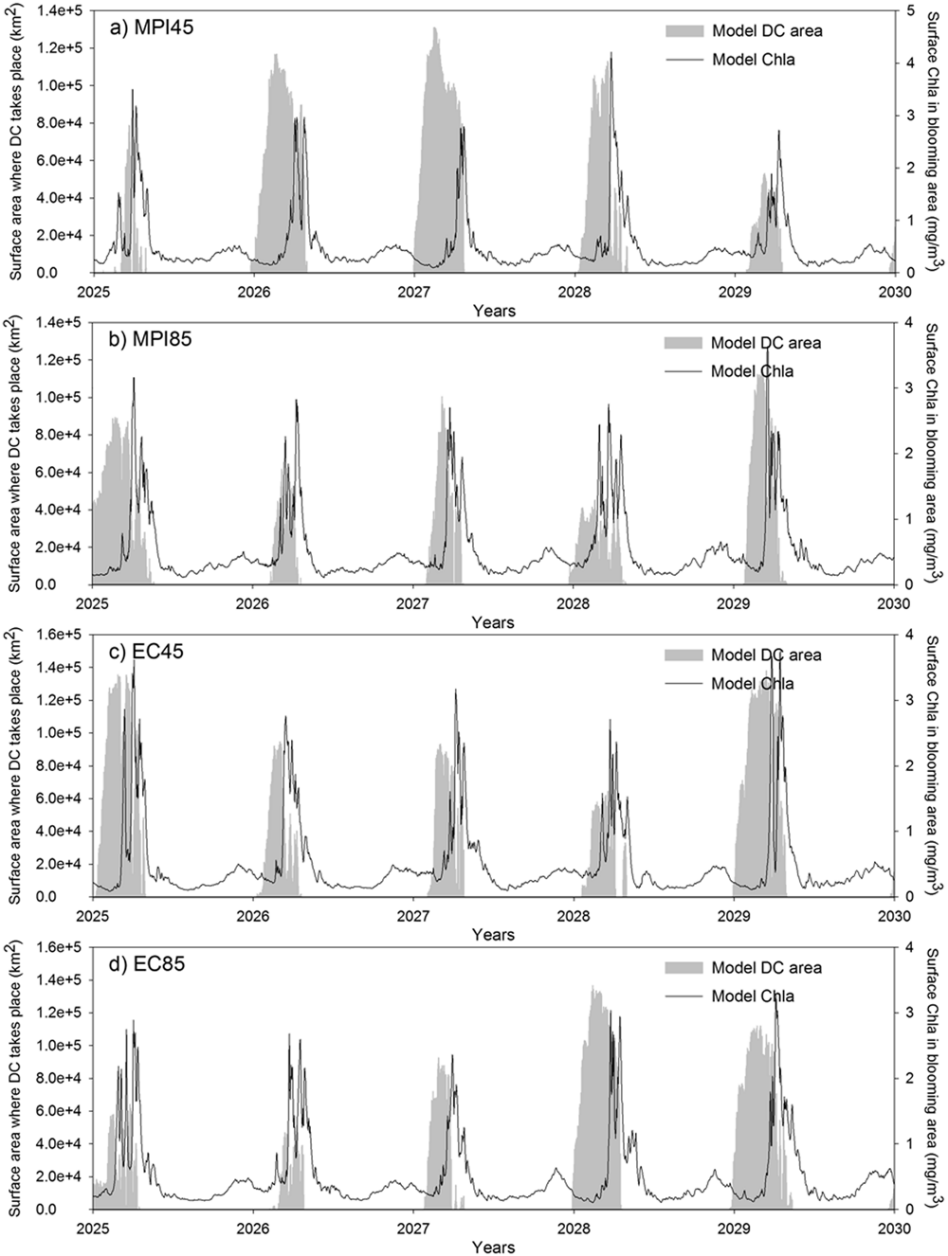
Time series of simulated area where DC takes place (gray bars) and simulated surface chlorophyll*-a* (Chla) (black line) for the different individual scenarios. (**a**) MPI at RCP4.5. (**b**) MPI at RCP8.5. (**c**) EC-Earth at RCP4.5. (**d**) EC-Earth at RCP8.5.

The interannual time-series of DC area and surface Chla (Fig. 5) does not show the same level of coherence observed for the present-day situation (Fig. 3b). Indeed, the scatter plot of accumulated area where DC takes place against the maximum intensity of the Chla bloom (Fig. 4b) does not show any significant correlation, in opposition to which was observed for the present-day (Fig. 3c).

**Figure 6:**
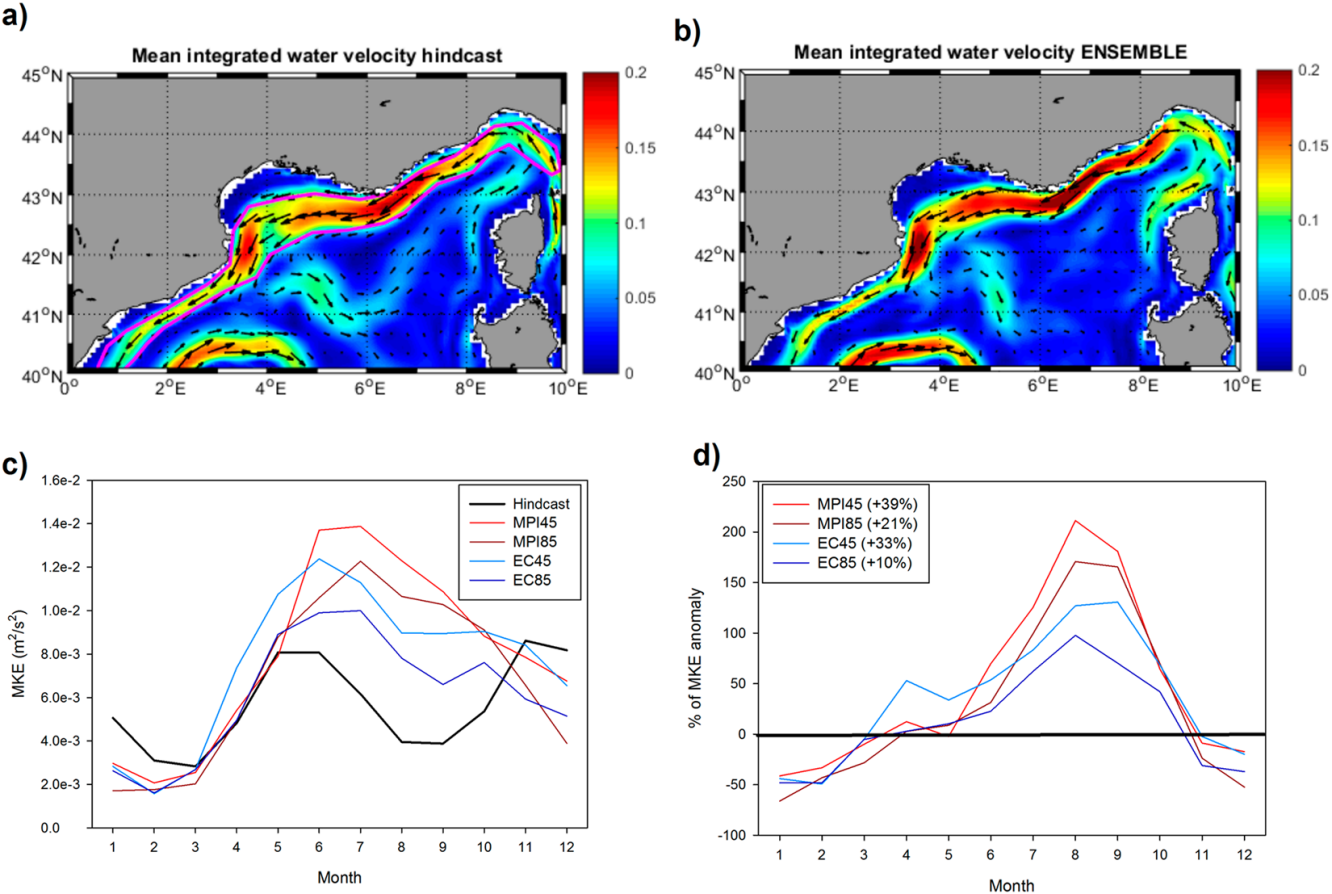
(**a**) Mean surface water circulation for the hindcast simulation (2010–2015). Background color indicate the mean velocity (m s-1) while the black arrows show the direction. The magenta line indicate the region occupied by the Northern Current (see text for details). (**b**) Same as a) but for the ENSEMBLE simulation. (**c**) Mean kinetic energy of the Northern Current (the magenta area in panel a) for the hindcast (black line) and the four different scenarios (colored lines). (**d**) Relative anomaly (in %) of the mean kinetic energy of the North Current in the different scenarios with respect to the hindcast simulation. Maps were created by the authors using MATLAB software vR2014b (https://it.mathworks.com/products/matlab/matlab-graphics.html).

To try better understand why DC seems to be less relevant for the future spring blooms in the region, we analyzed the dynamic conditions of the surface layer of the NW Mediterranean in the present-day and in the scenarios (Fig. 6). For the hindcast (2010–2015) the simulated surface currents show the presence of the very strong, slope-attached Northern Current (NC) flowing from the northeast to the southwest of the area^5^. Also, the quasi-permanent cyclonic circulation could be observed in the Gulf of Lion region, which is deemed as one of the main necessary elements to create deep convection in the area^10^. For the ENSEMBLE simulation (Fig. 6b) the mean intensity of the NC increases while no major changes in the open-sea circulation are apparent.

If we compute the mean kinetic energy (MKE) of the NC (i.e., in the area defined by the magenta lines in Fig. 6a) it could be observed that it has a certain seasonality (black line, Fig. 6c), with maximum intensities at the end of the spring and at autumn and a relative minimum during early winter and summer in agreement with previous observations^67^. For the different scenarios (colored lines, Fig. 6c) the MKE of the NC does not change much during winter but it increases quite dramatically during the stratification period. Indeed, in all scenarios the mean annual MKE of the NC increases between 10% and 39% with larger positive differences between July and November and negative (smaller) differences for December, January and February (Fig. 6d).

**Figure 7:**
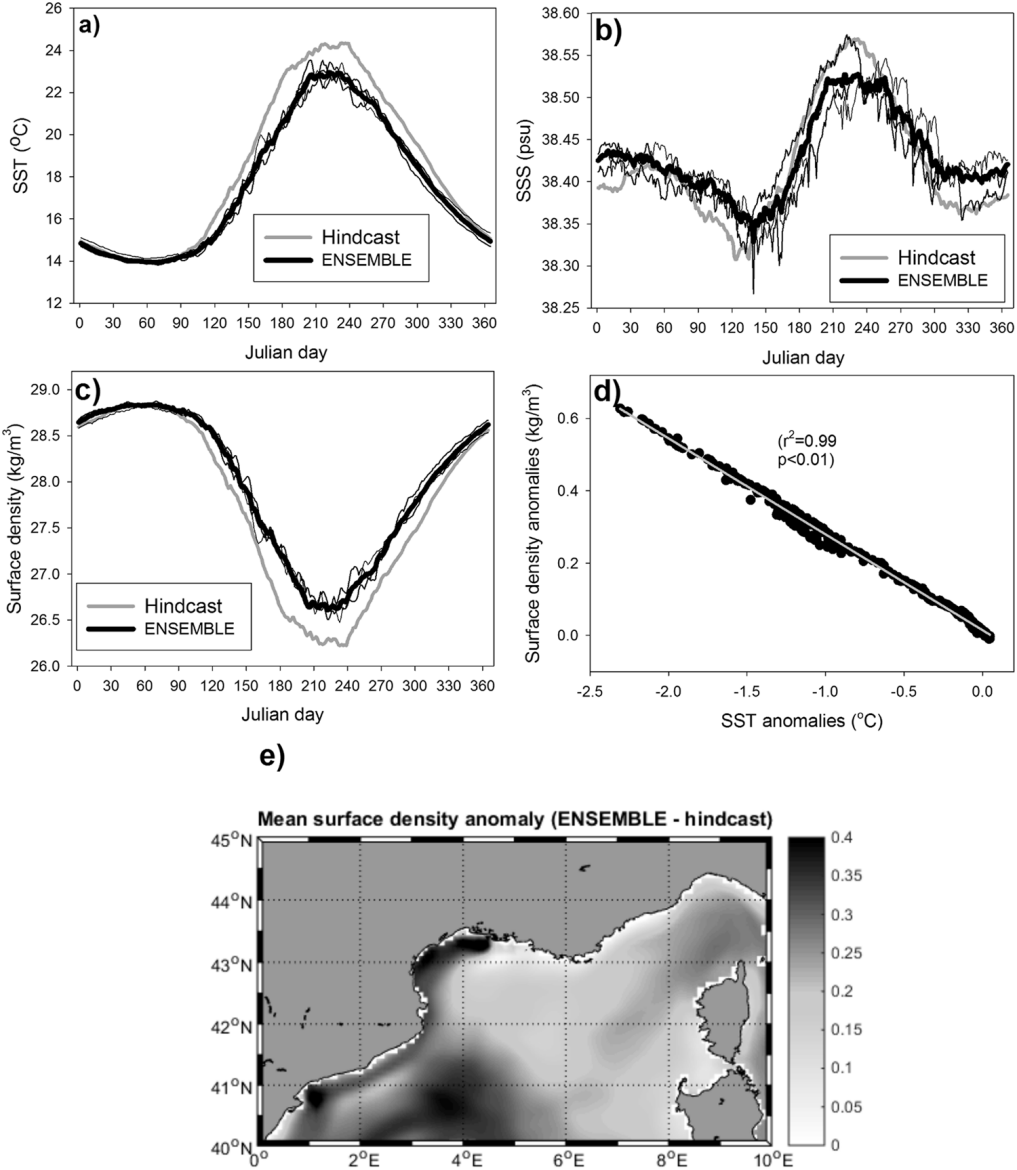
(**a**) Climatological sea surface temperature (SST) in the NW Mediterranean for the hindcast (thick gray line), for the ENSEMBLE simulation (thick black line) and for the individual members of the ENSEMBLE (thin black lines). (**b**) The same as a) but for sea surface salinity (SSS). (**c**) The same as (a) but for surface density. (**d**) Scatter plot of the anomalies (ENSEMBLE – hindcast) of SST versus surface density. The linear regression is shown as a gray line. (**e**) Mean surface density anomaly (ENSEMBLE – hindcast) map. Maps were created by the authors using MATLAB software vR2014b (https://it.mathworks.com/products/matlab/matlab-graphics.html).

Simulated thermohaline properties of the NW Mediterranean region are also changing in the different scenarios (Fig. 7). Surprisingly, sea surface temperature (SST) is simulated to decrease in the horizon 2030 for all members of the ENSEMBLE (Fig. 7a) with larger decrease in summer temperatures and smaller differences for winter. The behavior of the sea surface salinity (SSS) is more complicated (Fig. 7b) as there are periods when the future scenarios show larger SSS values and other when SSS is lower. Also the inter-ENSEMBLE spread is larger for SSS than for SST. Regarding surface density (Fig. 7c), all members of the ENSEMLE simulate higher values for the future than the hindcast run, with larger differences happening in summer. Actually, SST and surface density changes seems to be related and, indeed, the scatter plot of SST anomalies versus surface density anomalies (Fig. 7d) shows a high correlation coefficient. This is indicating that SST changes are responsible for the density anomalies (at least more than SSS changes). Finally, Fig. 7e shows the map of the mean surface density anomaly (ENSEMBLE – hindcast) for the NW Mediterranean. All values are positive (notice the gray scale) with larger anomalies happening in the southern part of the region and smaller differences in the central regions.

The atmospheric conditions over the NW Mediterranean Sea seem to change with respect to the present day situation (Table 2). From the three atmospheric variables that have been shown to influence more the simulation of the thermohaline properties of the surface Mediterranean Sea^66^, it seems that the major change happens in the meridional wind intensity (v10) that increases in average around 22% in the future simulations. The other two atmospheric variables (zonal wind (u10) and air temperature (t2)) show very small (and negative) anomalies (Table 2).

**Table 2:** Mean values of the main atmospheric variables for the different simulations. u10: zonal wind velocity at 10 m (m/s); v10: meridional wind velocity at 10 m (m/s); t2: air temperature at 10 m (^o^C).

Simulation	u10 (m/s)	v10 (m/s)	t2 (°C)
Hindcast	1.20	−1.11	17.9
MPI 45	1.21	−1.59	17.2
MPI 85	1.07	−1.37	17.9
EC 45	1.07	−1.14	17.9
EC 85	1.09	−1.23	17.9
Mean difference (ENSEMBLE – hindcast)	−0.09 **(−7.5%)**	−0.23 **(+21.7%)**	−0.2 **(−1.12%)**

To test the effect that an increase of wind forcing would have on our model simulations we performed another 16 year simulation (2000–2015) in which the wind forcing from the ERAin reanalysis was changed over the NW Mediterranean according to the mean anomalies shown in Table 2. DC in this simulation (Fig. S2a) starts earlier and lasts longer than in the standard hindcast, in a similar fashion as happen in the future scenarios (Fig. 4a). However, maximum extension of DC area is lower than in the standard hindcast. With this modified winds the Chla bloom lasts longer and reaches higher maximum values (Fig. S2a) similar to the modifications simulated in the scenarios (although the attained maximum are not as high as the one observed in Fig. 4a). Moreover, and as happened in the future scenarios, in this hindcast simulation the relationship between annual DC extension and spring bloom intensity is lost (Fig. S2b). Finally, the mean surface currents for this simulation (Fig. S2c) show a strengthening of the NC (as in the ENSEMBLE simulations, Fig. 6b) but also some large differences in the open-sea regions, especially a strong south-westerly current in the Gulf of Lion region not simulated in any of the previous model runs.

## Discussion

The temporal and spatial pattern of the MLD in the NW Mediterranean shown in Fig. 2 indicates that the model is able to simulate the main characteristics of the DC in the region. The climatological cycle indicates that DC is a winter process^10^ when the overall MLD in the region is larger than 250 m. This same figure indicates the strong variability of the DC annual cycle^33^ as the error bars during the winter (convective) months are quite large while they become much smaller during the stratification period.

Also, the spatial distribution of the winter MLD agrees reasonably well with previous reports; there is a main convection region in the off-shore waters of the Gulf of Lion^32^ and a less frequent area in the Ligurian Sea^65^. In previous modelling works, this latter, more elusive area for DC has been difficult to simulate^36^ due to too-strong stratification of the water column. However, it seems the MF is able to pick up this characteristic of the DC events in the NW Mediterranean.

Indeed, the MF used here has been shown to be able to reproduce the main open-sea DC regions in the Mediterranean Sea, such as the southern Adriatic and the Crete convection regions^59^. It cannot, however properly represent DC events happening closer to the coast, such as those in the northern Adriatic^68^ due to its relatively coarser spatial resolution both in the oceanic compartment (~9 × 9 km) and in the atmosphere (typically between 12 × 12 km for the CCLM and 80 × 80 km for the ERAin). Also, the air-sea interaction routine in the MF (see details in Methods) ignores the feedback of the SST changes on the air temperature which can induce some deviations from the real values. More sophisticated two-way (atmosphere-ocean) coupled models of the entire basin have already been shown to be able to represent DC in different regions of the Mediterranean Sea^36^. However, this approach is still not advanced enough to provide consistent predictions of SST without the need of data assimilation to constrain model drift. This is not an issue when independent data do exist to constrain the model, but it could pose a problem when doing predictions outside the observed time-frame (i.e., when doing future scenarios simulations).

There are, also, other important elements not included in the MF such as the effects of the surface waves on the air-sea heat fluxes. It has been shown that waves could be important for the transference of energy within the water column, affecting, thus, the quantity and quality of deep water created during the convection and its posterior spread into the basin interior^69^. Another potentially shortcoming of the MF is not considering the tides. Even if the tidal range within the Mediterranean is minimal, it has been shown^70^ that tidal mixing in the Gibraltar Strait could influence the winter ‘preconditioning’ phase in the NW Mediterranean by altering the thermohaline properties of the incoming Atlantic waters. All these missing elements could make the MF to not exactly represent the extension, intensity and development pattern of single DC events but, from the comparisons shown above, our approach could suffice for making long-term, climate-like simulations, coming at a much lower computational cost than more sophisticated approaches.

The strong and significant correlation between the mean spatial map of winter MLD (Fig. 2b) and of the spring surface Chla from MODIS (Fig. 1b) shown in the scatter of Fig. 2d is a first order indication that, indeed, the spring bloom is controlled by the mixing and fertilization originated during the DC events^16,37,39,71^. We must, however, also consider that the general cyclonic circulation on the open-sea regions of the investigated area^10,11^ could help to concentrate and maintain the phytoplankton within the same area, contributing, thus, to the spatial coincidence between DC and Chla.

However, the hypothesis that DC strength is modulating the bloom intensity is further supported by the coherence shown in Fig. 3b between the annual DC and the surface Chla during the 16 years of the hindcast simulations. The significant statistical relationship found between the integrated annual convection and the magnitude of the spring bloom (Fig. 3c) also serve as additional proof of the strong impact the DC has on the development and strength of the seasonal Chla peak as suggested in previous works^4,41,72,73^.

In summary, for the present day conditions it seems quite evident that the position, extension and timing of the winter DC events are one of the major drivers of the spring bloom in the NW Mediterranean Sea as repeatedly reported by many authors^73,74^. This strong connection between DC strength and open-sea phytoplankton blooms intensity also agrees well with the recent findings by Macias *et al*.^62^ who reported how the river-borne fertilization carried into the Gulf of Lion region is mostly affecting the eutrophication status of the coastal waters, with minor impacts in the regions beyond the continental shelf slope, where internal oceanographic processes regulate primary production.

It is also worth mentioning the quite good correlation between the Chla levels computed from the different satellite products and those simulated by the MF at both seasonal scale (Fig. 3a) and using the individual 8 days composites (Fig. 3b) during the entire hindcast period. The capability of the MF to represent surface Chla levels at monthly time-scale was already proved in Macias *et al*.^59^ but the high frequency comparison shown in here demonstrates that the system is able to capture the fine-scale details of Chla dynamics, at least in the analysed NW Mediterranean area. The seasonal cycle depicted by both model and satellite Chla represents the typical from temperate ocean with a spring bloom, followed by summer oligotrophy and a less intense bloom in autumn^20,21^.

It is also worth mentioning that in the Mediterranean Sea satellite-based Chla estimates could not be accurate in several instances, such as during the stratification period when sub-surface Chla accumulations are very common^59^, during the specific moments when DC happens as large Chla values could be spread down to several hundred meter depth^75^ or into some coastal areas^76^. However, for the spring time and for the analysed area, previous modelling works have shown that surface Chla concentration is quite homogeneous in the first 100 m^59^ so, for the purposes of the comparisons shown in here, satellite data could be considered accurate enough.

Simulated interannual variability of DC is quite large as shown in Fig. 3b (grey bars) and in agreement with previous reports^33,36,51^. Especially striking are the very reduced DC during winter 2000–2001 as reported in previous works^29^ and the almost total lack of DC in winter 2006–2007 (also shown by Somot *et al*.^36^) which was followed with the weakest spring bloom of the entire hindcast period (colour lines in Fig. 3b). Also, the much larger DC events documented in winters of 2004 and, particularly, 2005^51^ are well captured in our simulations.

The fact that the MF is able to simulate the main mesoscale circulation features of the NW Mediterranean (Fig. 6a) such as the Northern Current^5^ and the presence of the cyclonic gyre in the Gulf of Lion^10^ is probably one of the main reasons why DC is properly simulated. It has been recurrently described how the winter deepening of the ML in this region happens, principally, within the area defined by the cyclonic gyre that uplift the isopycnals, isolates the water mass and exposes it to the action of the atmospheric forcing^12^. Additionally, sub-mesoscale processes originating at the Northern Current^67,77^ help to create instabilities in the ‘mixing’ region, contributing to the DC events^23,78^.

Overall, the reasonable capability of the MF to represent the DC events and the associated Chla blooms makes this system an appropriate tool to study potential changes for the mid-term future (~2030) under climate change scenarios. This time horizon is not the typical used in climate-change studies, that focus more into the long-term future (at least 80–90 years) because at that time-scale it is easier to separate the climate change signal from the natural variability^58^ and to find differences between the different emission scenarios^79^. However, we decided to centre our attention to this 10–15 years horizon as this is a time-frame more sensible and meaningful for managers and stake-holders. The good agreement of the results obtained in the present work for the different scenarios shows that, even at this short time-scale, assessments of the impacts of climate change on our marine ecosystems could be done with current-generation climate models, always using due caution given the inherent uncertainties in climate projections.

The main conclusion from the ENSEMBLE scenarios is that, in the future, the strength and duration of the annual DC increases. This result agrees with the work of Ulses and colleagues^38^ who also simulated an increasing DC in the region during the past decades related with an increase in surface density and, hence, to a decrease in vertical stratification and water column stability. However, those authors found the increase in surface density linked to an increase in SSS while in our scenarios it is connected to a decrease of SST (Fig. 7d). There are also other works^53,80–82^ where DC intensity in the region is simulated to decrease in the future linked with a surface density decrease due to SST increases. In these simulations, vertical stability of the water column increases, making mixing and DC harder. With a similar prediction for the future hydrological conditions in the region, Herrman *et al*.^56^ found a decrease in its overall primary production.

The same MF used here simulated, for the end of the 21^st^ century, a negative anomaly on surface density linked to a SST increase^61^ in this region. Also, primary productivity in the area is predicted to decrease in that scenario. Although vertical mixing and, hence, DC is not computed in that previous work, based on the surface anomalies it is expected that DC events are reduced in the area for the considered time-horizon. It is, precisely, on this time-frame where the main difference between the previous paper^61^ and the present contribution lays. If the time-series of basin-wide SST presented in Fig. 2a of Macias *et al*.^61^ are examined, it could be clearly seen that for the time-frame considered here (~2030) there are no significant differences between the diverse models/RCPs combination and neither with respect to present-day values. This lack of difference in SST values is, most likely, related with the small increase in air temperature predicted by the CGMs in the Mediterranean region for this time-frame which is only evident after ~2050 as the SST evolution in that figure shows.

Indeed, in the time-horizon considered here, air temperature over the NW Mediterranean remains almost unchanged with respect to its actual value (−1.12%, Table 2) while wind intensity increases (specially the meridional component, ~22% Table 2). This increasing wind forcing favours vertical mixing and, hence, decreases SST (see Fig. 7a). As shown by the highly significant correlation in Fig. 7d, the decrease in SST drives the increase in surface density, which reduces vertical stratification. This decrease in vertical stratification makes mixing more efficient and DC more plausible, explaining why the DC period and the maximum area where DC happens are increased in the ENSEMBLE simulations (Figs 4a and 5).

The delay in the termination of the spring bloom in the future (red line, Fig. 4a) could be related with the longer DC period. More nutrients are delivered to the surface during more time in the scenarios making phytoplankton grow during a longer period. However, this phenological change in DC events does not explain the increase in phytoplankton biomass during the stratified period and the lack of correlation between the intensity of the DC and the phytoplankton bloom intensity (Fig. 4b).

As seen in Fig. 6, the MKE in the region increases in the future scenarios, especially during the stratification period (i.e., when DC is no longer important). The NC has been shown to constitute a significant source of nutrients through vertical mixing and horizontal advection for the pelagic ecosystem of the region^61,83^ so a stronger NC (especially in summer) could be the main cause for the enhanced Chla levels during this period of the year (Fig. 4a).

It might also be that the increasing importance of mesoscale activity on delivering nutrients to the surface layer in the future scenarios with respect to the present-day conditions is one of the reasons why in the future there is no relationship between the magnitude of the annual DC and the intensity of the bloom (Fig. 4b). This hypothesis seems to be confirmed by the results of the ‘wind modified’ hindcast simulation, as in Fig. S2b there is no significant correlation between DC intensity and annual phytoplankton bloom.

Indeed, this simulation performed with the modified wind intensity during the hindcast period seems to confirm the crucial role wind forcing change has on the results for the different scenarios (as also shown by Estournel *et al*.^43^ and Myers *et al*.^46^). The simulated DC annual cycle and surface Chla dynamics (Fig. S2a) with the modified wind intensity are somewhere between the present-day conditions and the ENSEMBLE results shown in Fig. 4a although both the DC maximum extension and the bloom intensity are lower than in the scenarios. The reason for these mismatches could be related with the different circulation pattern simulated with the modified winds (Fig. S2c) as in this case the cyclonic cell in front of the Gulf of Lion (one of the key features to create DC) is disrupted by a strong south-easterly current not present in any of the previous simulations (see Fig. 6a,b).

In conclusion, our modelling simulations seem to indicate that in the near future (~year 2030) the main control mechanisms of primary production in the NW Mediterranean Sea could likely change. From a system where winter DC process seems to be major driver of open-sea plankton seasonality, the ENSEMBLE simulation indicates a shift to a condition where mesoscale activity has a more crucial role for nutrient delivery into the euphotic layer. This change seems to cause a phenological change of plankton seasonality with the spring bloom happening later in the year and lasting longer, higher phytoplankton biomass present during the stratification period and an earlier and more intense autumn bloom. Such changes in the base of the food web could have implications for the entire ecosystem, as this NW Mediterranean region is a very important feeding and nursery ground for many commercially-important species^84,85^ and for ecologically-relevant higher trophic levels^86,87^. We have also shown how the MF here employed could provide useful information on a time-scale that’s relevant for managers and policy-makers, further stressing the potentialities of this tool to be used as policy- and decision-making support.

## Methods

### Satellite data

8 days composites of Chla obtained from the Aqua MODIS sensor (Moderate Resolution Imaging Spectroradiometer) with spatial resolution 4 km were downloaded from the NASA web server (accessible at https://oceandata.sci.gsfc.nasa.gov/MODIS-Aqua/) covering the period January 2003–December 2015. A detailed description of the Aqua MODIS mission can be found in https://oceancolor.gsfc.nasa.gov/data/aqua/.

An alternative satellite product used here are the Chla 8 days composites from the SeaWiFS sensor with 9 km of spatial resolution available at https://oceandata.sci.gsfc.nasa.gov/ SeaWiFS/. SeaWiFS datasets provide Chla maps for 2000 to 2002, which are years not covered by MODIS Aqua, and are available to December 2010. A detailed description of the SeaWiFS mission is in https://oceancolor.gsfc.nasa.gov/data/seawifs/.

### Modelling framework

The oceanic component of the MF is composed by two coupled models, a hydrodynamic model based on GETM^88^ and a biogeochemical model based on ERGOM^89^. A detailed description of the GETM equations could be found in^90^ and at http://www.getm.eu. Our implementation for the Mediterranean Sea has a horizontal resolution of 5′ × 5′ (~9 × 9 km) and includes 25 vertical sigma-layers. Model bathymetry was built using ETOPO1 (http://www.ngdc.noaa.gov/mgg/global/) database while initial thermohaline conditions were created by using the Mediterranean Data Archeology and Rescue-MEDAR/MEDATLAS database (http://www.ifremer.fr/medar/). The same MEDAR/MEDATLAS data was used to create the boundary conditions for the model at the Strait of Gibraltar where monthly climatological vertically-explicit values of salinity and temperature are imposed. No horizontal currents are explicitly prescribed at the open boundary.

GETM is forced at the surface every 6 hours by the following atmospheric variables: wind velocity at 10 meters, air temperature at 2 m, dewpoint temperature at 2 m, cloud cover and atmospheric pressure at sea level. Atmospheric data could come from reanalysis or from atmospheric models (see details below). In both cases, bulk formulae^58^ are used to calculate the corresponding relevant heat, mass and momentum fluxes between atmosphere and ocean by combining atmospheric variables with surface ocean information such as SST and current velocities. The effective heatflux (to/from the ocean) is based on the difference in air temperature from the atmospheric data and actual SST from GETM. On a similar way, the effective surface stress is computed using the difference between the wind velocity and the surface current velocity (see scheme in Fig. S3).

The ocean model includes 53 rivers discharging along the Mediterranean coast. Values for river discharges were derived from the Global River Data Center (GRDC, Germany) database while inorganic nutrient loads (nitrate and phosphate) of freshwater runoff were obtained from^91^. It must be stressed that rivers’ conditions (flow and chemical composition) are kept invariable for the future scenario runs described below. This way the effects of a changing atmospheric forcing on the marine ecosystem could be isolated and quantified (as it was done in^61^).

A continuous and small atmospheric input of nitrate, phosphate, and ammonium (equivalent to their climatological mean) is imposed in the entire model domain^63^: nitrate ~8.0e^−2^ mmol/m^2^d and ammonium ~4.0e^−2^ mmol/m^2^d, while phosphate was set at ~1.2e^−3^ mmol/m^2^d assuming a N:P in the atmospheric deposition ~100^92^.

GETM is coupled online to the MedERGOM biogeochemical model^59,60^ by using the Framework for Aquatic Biogeochemical Models (FABM, https://sourceforge.net/projects/fabm/)^93^. MedERGOM is a modified version of the ERGOM model^89^ specifically adapted to represent the conditions of the pelagic ecosystem of the Mediterranean Sea. Briefly, MedERGOM incorporates three phytoplankton functional types (‘diatom-like’, ‘flagellates-like’ and ‘cyanobacteria-like’), two major nutrients (nitrate and phosphate), one zooplankton compartment and detritus. To get a more comprehensive description of this model the reader is referred to^59^ and^61^. Biogeochemical initial and boundary conditions are computed from the World Ocean Atlas database (www.nodc.noaa.gov/OC5/indprod.html).

### Atmospheric forcings

The MF described above incorporates the atmospheric component as an integral part of the RESM. As commented, this atmospheric compartment could be either a database (*i*.*e*., a reanalysis) or inputs from atmospheric models. For the present contribution we perform two types of simulations, one aiming to simulate the present-day conditions of the system (hindcast) and another exploring future scenarios.

The hindcast simulation covers the period 2000–2015 and uses the European Centre for Medium-Range Weather Forecasts (ECMWF) ERAin database. ERAin provides relevant information on atmospheric conditions at a horizontal resolution of 80 km and have been shown to create reasonable ocean surface conditions in the Mediterranean Sea when used in combination with GETM^58^.

For the future scenarios, GETM is forced by the atmospheric conditions generated by a Regional Climate Model (RCM) called COSMO-CLM (CCLM; http://www.clm-community.eu/), in the framework of the EURO-CORDEX initiative (http://www.euro-cordex.net/). This RCM is forced at the boundaries by two Global Circulation Models (GCMs), namely EC-Earth and MPI-ESM-MR included in the CMIP5 (Table 3). The RCM spatial resolution is 0.11°. For each GCM two emission scenarios as defined by IPCC are considered; RCP4.5 and RCP8.5^94^. Hence, a total of four member ensemble runs are analysed in this work. Continuous simulations from 2015 to 2031 are performed with each member of the ENSEMBLE. The comparisons with the hindcast simulations shown below are done by computing mean conditions in the scenarios for the period 2027–2031 (*i*.*e*., a five year period centred on year 2030).

**Table 3:** Institutes/modelling groups providing the atmospheric model data used in the present contribution.

Modelling group	Driving model name	Emission scenarios
ECEARTH consortium	EC-EARTH	rcp 4.5/rcp 8.5
Max-Planck-Institut für Meteorologie (Max Planck Institute for Meteorology)	MPI-ESM-MR	rcp 4.5/rcp 8.5

As a previous step to the scenario runs and as concluded in^66^, a bias-correction of the most relevant atmospheric variables in the RCM (air temperature, cloud cover and wind intensity) should be performed. As shown in^66^, forcing the ocean model of the MF with the atmospheric variables provided by the RCM realizations induce a severe underestimation of simulated SST for the present-day, so a pre-processing (‘correction’) of such variables is needed. The basic principle of the bias-correction technique consisted in finding a transfer function that allows matching the cumulative distribution functions (CDFs) of modelled and observed data^95–97^. In our study, spatially-averaged values of the observed and model atmospheric variables over the entire Mediterranean Sea basin were used, so no spatially explicit correction was applied. A detailed description of the bias-correction technique and evaluation over the present climate could be found at Macias *et al*.^66^. We need to stress that, as mentioned before, the rivers’ conditions (flow and water chemical quality) are kept unchanged in the different scenario runs^55^.

### Data availability

All data used for the present contribution could be requested through the Environmental Marine Information System (EMIS) of the EU Commission (http://mcc.jrc.ec.europa.eu/emis/).

## Electronic supplementary material

Supplementary information
