# Supplementary material for: Deep winter convection and phytoplankton dynamics in the NW Mediterranean Sea under present climate and future (horizon 2030) scenarios
Source: Sci Rep. 2018 Apr 26;8:6626. doi: 10.1038/s41598-018-24965-0 (PMC5919909; doi:10.1038/s41598-018-24965-0)
Supplement: Supplementary file 1 — Supplementary information [file 41598_2018_24965_MOESM1_ESM.pdf]

# SUPPLEMENTARY INFORMATION FOR:

# Deep winter convection and phytoplankton dynamics in the NW Mediterranean Sea under present climate and future (horizon 2030) scenarios

Diego Macias, Elisa Garcia-Gorriz, Adolf Stips

European Commission, Joint Research Centre, Directorate D- Sustainable Resources, Via E. Fermi, 21027, Ispra (VA), ITALY

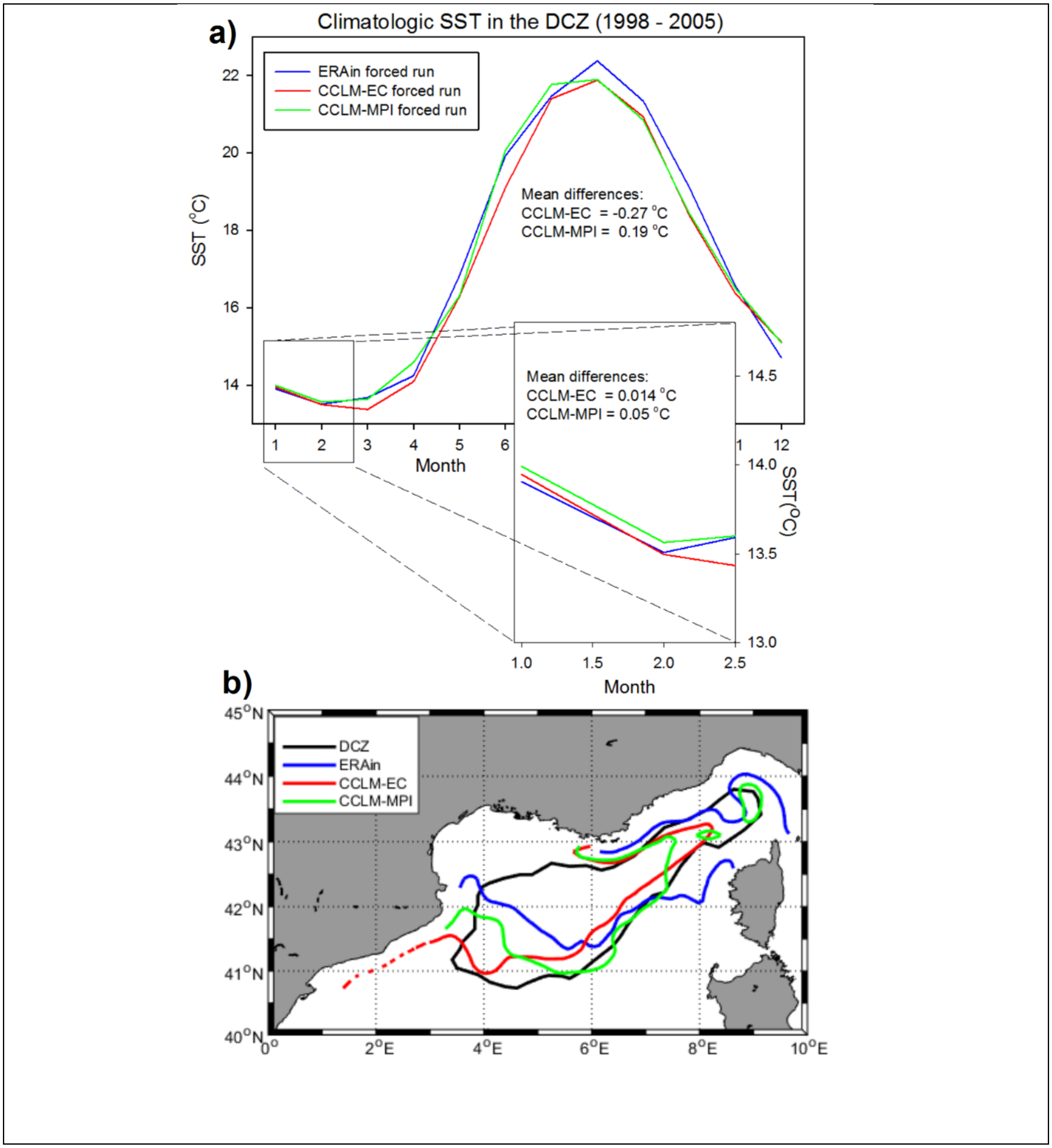


Figure S1. a) Simulated climatological SST in the DCZ (defined from Fig. 2b, MLD=650m) during the period 1998 - 2005 using ERAin forcing (blue line), and CCLM with the two RCMs, EC-Earth (red line) and MPI (green line). b) DCZ defined from Fig. 2b (black line) and using the isotherm 13.7 in the winter months form the different model runs in the 1998 – 2005 period (blue: model forced with ERAin; red model forced with CCLM-EC; green: model forced with CCLM-MPI). Map in panel b) was created using MATLAB software vR2014b (https://it.mathworks.com/products/matlab/matlab-graphics.html)

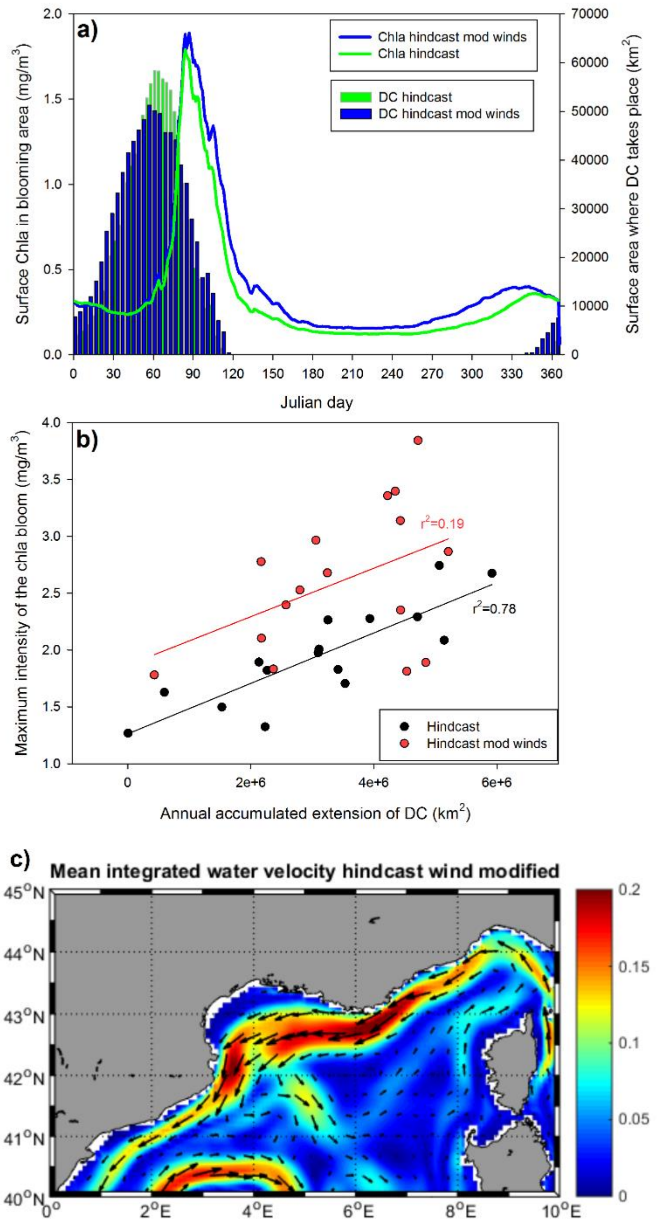


Figure S2. Results of the simulation for the hindcast period (2000 – 2016) using the modified winds computed with the anomalies shown in Table 1. a) Climatological cycle of DC (blue bars, modified winds; green bars, 'normal' hindcast) and surface chlorophyll (blue line, modified winds; green line, 'normal' hindcast). b) Scatter plot of accumulated area where DC occurs versus the maximum intensity of the spring bloom (red dots and line, modified winds; black dots and line, 'normal' hindcast). c) Mean surface currents for the modified wind hindcast simulation. Map in panel c) was created using MATLAB software vR2014b (https://it.mathworks.com/products/matlab/matlab-graphics.html)

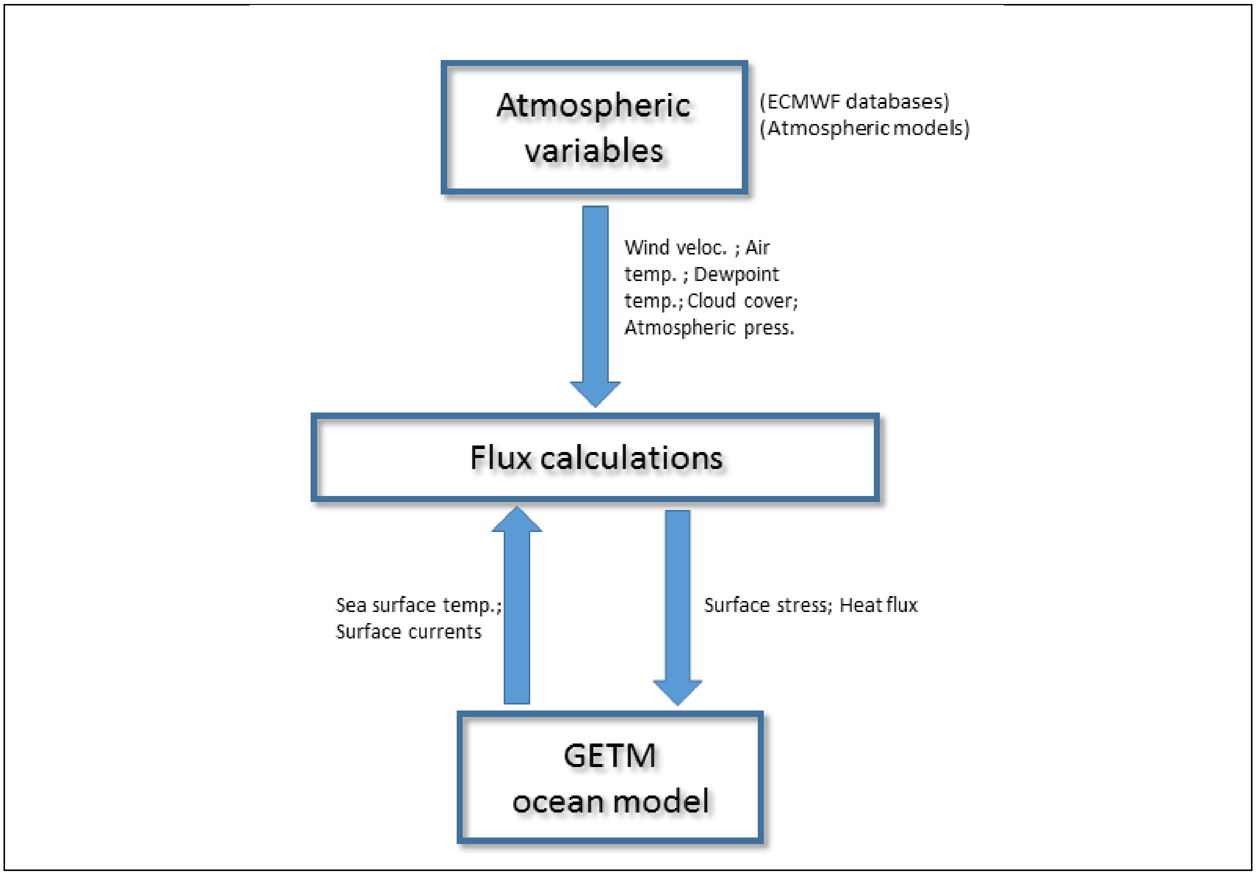


Figure S3. Schematic representation of the atmosphere-ocean interactions in the Modeling Framework.
